# Does Chronic Idiopathic Dizziness Reflect an Impairment of Sensory Predictions of Self-Motion?

**DOI:** 10.3389/fneur.2013.00181

**Published:** 2013-11-08

**Authors:** Jörn K. Pomper, Lena Gebert, Matthias Fischer, Friedemann Bunjes, Peter Thier

**Affiliations:** ^1^Department of Cognitive Neurology, Hertie-Institute for Clinical Brain Research, University of Tübingen, Tübingen, Germany; ^2^Department of Psychiatry and Psychotherapy, University Hospital Tübingen, Tübingen, Germany

**Keywords:** dizziness, vertigo, chronic idiopathic dizziness, phobic postural vertigo, efference copy, smooth pursuit, self-motion perception, Filehne illusion

## Abstract

Most patients suffering from chronic idiopathic dizziness do not present signs of vestibular dysfunction or organic failures of other kinds. Hence, this kind of dizziness is commonly seen as psychogenic in nature, sharing commonalities with specific phobias, panic disorder, and generalized anxiety. A more specific concept put forward by Brandt and Dieterich (1) states that these patients suffer from dizziness because of an inadequate compensation of self-induced sensory stimulation. According to this hypothesis self-motion-induced reafferent visual stimulation is interpreted as motion in the world since a predictive signal reflecting the consequences of self-motion, needed to compensate the reafferent stimulus, is inadequate. While conceptually intriguing, experimental evidence supporting the idea of an inadequate prediction of the sensory consequences of own movements has as yet been lacking. Here we tested this hypothesis by applying it to the perception of background motion induced by smooth pursuit eye movements. As a matter of fact, we found the same mildly undercompensating prediction, responsible for the perception of slight illusory world motion (“Filehne illusion”) in the 15 patients tested and their age-matched controls. Likewise, the ability to adapt this prediction to the needs of the visual context was not deteriorated in patients. Finally, we could not find any correlation between measures of the individual severity of dizziness and the ability to predict. In sum, our results do not support the concept of a deviant prediction of self-induced sensory stimulation as cause of chronic idiopathic dizziness.

## Introduction

Chronic dizziness is a highly prevalent complaint in the daily practice of general medicine and neurology (2, 3). Similar to acute disease states it can indicate a vestibular dysfunction. This is particularly suggestive if dizziness is accompanied by circumscribed illusions of motion of oneself or the surroundings, which is a symptom combination captured by the term vertigo. In many cases, however, an underlying vestibular or non-vestibular organic dysfunction cannot be revealed with certainty. Several attempts have been made to find alternative explanations of this dizziness of unknown origin often referred to as to “idiopathic dizziness.” Although there are differences in the detailed diagnostic criteria and the pathophysiological mechanism implicated, common to most concepts is the considerations of three clinical hallmarks: (1) a non-vertiginous character of dizziness, (2) above-normal levels of anxiety up to panic attacks and avoidance behavior, and (3) a strong situational dependency of dizziness and anxiety (4–8).

The non-vertiginous character accompanied by anxiety, partly fulfilling the diagnostic criteria of anxiety disorders, in combination with other psychopathological conditions like depression or compulsive personality traits and the report of life events that preceded the onset of dizziness have been taken to suggest a psychological causation of chronic idiopathic dizziness. Concepts emphasizing this mechanism stand under headings such as “primary and secondary somatoform vertigo” or “chronic subjective dizziness” (7–9). Apart from psychodynamical explanations the most prominent principle adopted is classical conditioning. Any primary cause of dizziness is considered the unconditioned stimulus and the accompanied perception of motion the conditioned stimulus leading to the reaction of feeling dizzy (7). In principle, this learning process may also associate normal motion stimuli with the sensation of dizziness. In more extreme cases, even stimuli not related to motion may function as conditioned stimulus, leading to generalization of situational dependency and, as a consequence, increased anxiety and avoidance behavior. The strength of this putative mechanism is the explanation of situational dependency and its generalization. Its weakness, however, is that it does not explain the primary occurrence of dizziness and that it might apply to any other feeling as well. The specific nature of dizziness, which might be best described as a disturbance of perceptual stability of oneself relative to the external world is ignored.

A more cognitive, partly complementary approach centers on this disturbance of perceptual stability as the central feature of the subjective experience of dizziness. Any explanation of dizziness within this framework has to pinpoint a mechanism violating perceptual stability. Traditionally, thinking revolved around dysfunctions of the vestibular system with the possibility of more subtle changes in chronic idiopathic dizziness supposedly left unnoticed by routine tests of the vestibular system. A more modern view is based on the realization that perceptual stability is a product of multisensory integration of spatial information with contributions not only from the vestibular but also from the visual, the somatosensory, and the auditory systems (10). This implies that disturbances within each sensory modality and at different levels of processing are, in principle, capable of inducing dizziness. “Visual vertigo” and “space and motion phobia” are two examples of concepts within this framework that posit pathologic adjustments in the vestibular or visual modality in some patients with chronic idiopathic dizziness (11, 12).

**Figure 1:**
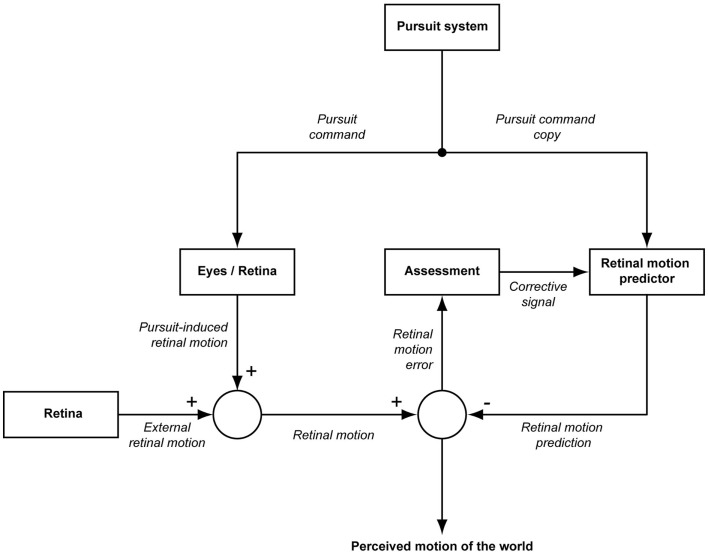
**Modified reafference principle adapted to smooth pursuit eye movements**. The pursuit system generates a motor command driving the eyes in order to yield a smooth pursuit eye movement (pursuit command). A copy of this pursuit command provides the input to a predictor of the sensory consequences of the eye movement in the sense of self-induced motion on the retina. The resulting prediction of self-induced retinal motion is compared with the actual retinal motion which is the sum of pursuit-induced and externally induced retinal motion. If the prediction of self-induced retinal motion is perfect and if there is no external motion, the output of the comparator will be zero, interpreted as stationarity of the visual background, i.e., the world. In the case of an optimal prediction, a non-zero output of the comparator indicates motion of the world. On the other hand, if background motion is perceived although the world can be assumed to be stationary, it must be the prediction that is flawed. In this case, the comparator output is used to recalibrate the predictor.

An intriguing extension of the multisensory concept of dizziness was suggested by Brandt and Dieterich for patients suffering from “phobic postural vertigo” (PPV), an entity, most probably largely congruent with chronic idiopathic dizziness, identified by the authors based on clinical commonalities among patients (1, 6). Brandt and Dieterich postulated an impaired prediction of the sensory consequences of one’s own actions in these patients as the mechanism leading to disturbed perceptual stability equivalent to dizziness. This idea was encouraged by patients’ reports of “illusory body perturbations” during eye, head, and body movements. It is based on well-established physiological concepts assuming that perceptual stability does not only require intact sensory inputs but also appropriate processing of information about own body movements in order to avoid the perception of motion illusions due to self-motion. A formal description of this principle was first presented by von Holst and Mittelstaedt who referred to it as the “Reafferenzprinzip” (the reafference principle) (13). The key feature of this principle is that a copy of the motor command, the “efference copy” is compared with the sensory feedback, annihilating contributions to the sensory stimulus that are a consequence of the movement of the observer, the “reafference,” thereby unveiling contributions from the external world (the “exafference”) (Figure 1). Actually, the scheme shown in Figure 1 describes the case of visual motion perception in the event of a particular form of self-motion, horizontal smooth pursuit eye movements. In this case, the afference is the sum of motion in the world and pursuit-induced motion of the retinal image. This scheme assumes that the efference copy is not a simple replica of the pursuit command as originally assumed by von Holst and Mittelstaedt but a prediction of the expected visual consequences of the pursuit eye movement, optimized based on prior experience (14). Provided the optimization of the prediction is efficient, the prediction will be able to perfectly eliminate the pursuit-induced reafference. As a consequence, only the true external motion is perceived while stationary objects are experienced stable.

The notion that disturbances of the reafference principle may lead to chronic dizziness is fully supported by the case of patient R.M. (15). This patient with bilateral parietooccipital lesions has been suffering from a complete inability to isolate the exafference due to an inability to deploy a viable prediction of pursuit-induced retinal image motion. This inability is experienced by R.M. as chronic disabilitating dizziness, not accompanied by any other complaint or deficit. Encouraged by this intriguing case, we were curious to test the hypothesis that also other patients with chronic idiopathic dizziness might suffer from deficits in the prediction of their visual consequences of their pursuit eye movements. As in previous studies we employed a psychophysical task that allowed us to measure the predicted retinal image motion during smooth pursuit eye movements.

## Materials and Methods

### Subjects

**Figure 2:**
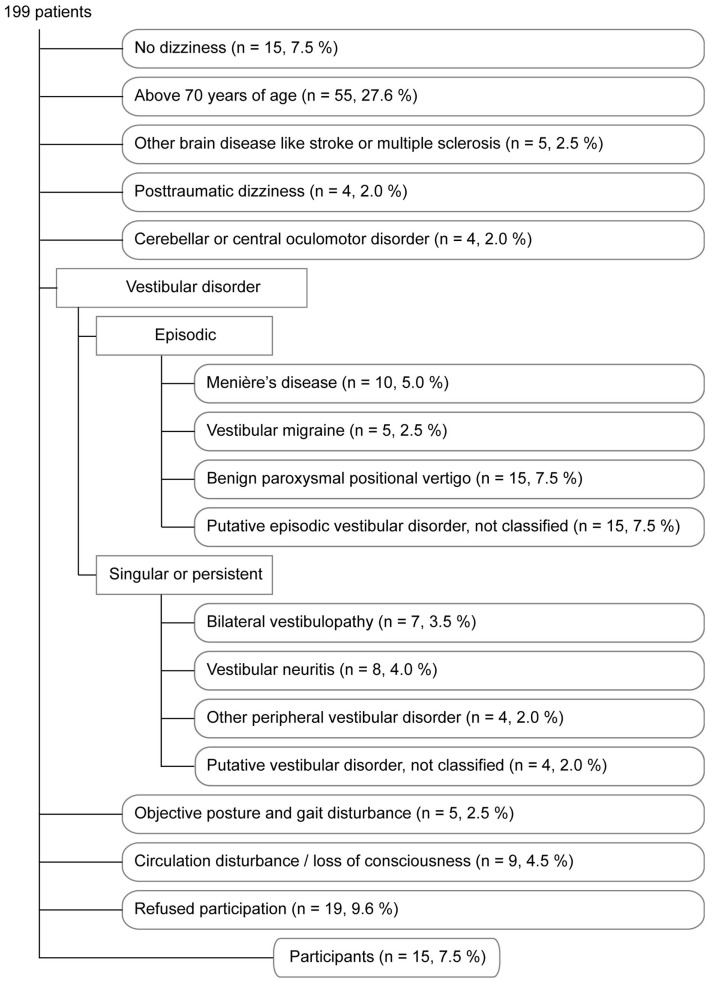
**The flow chart describes the reasons for excluding 184 out of 199 patients consecutively examined in our dizziness unit**. The order reflects the hierarchical process of exclusion. For example, a patient older than 70 years of age was excluded for this reason independent of the underlying disorder. In order to minimize the likelihood that patients with an organic disorder accompanied by dizziness were included into our study we regarded it sufficient to exclude and to classify patients once an exclusion disorder was clinically suspected.

Fifteen patients with chronic idiopathic dizziness were recruited in the dizziness unit of the Department of Cognitive Neurology (eight female, seven male; mean age 38.0 years, range 20–69). The diagnosis was based on the description of the complaints, normal neurological examination, and normal caloric testing. The following inclusion criteria were applied: (1) character of dizziness as assessed by offering the patients five descriptions out of which three needed to be affirmed for inclusion. The five German terms and their literal translations were “Schwankschwindel” (dizziness with the feeling of swaying), “Benommenheit” (light-headedness), “subjektive Unsicherheit beim Stehen und/oder Gehen” (subjective unsteadiness during standing and/or walking), “im Kopf stimmt etwas nicht” (something wrong in the head), and “Gefühl einer Gleichgewichtsstörung” (feeling of a disturbance of balance). (2) Dizziness had to be present during the last 3 months on at least 8 days a month. Patients were excluded if: (i) age was above 70 years because the likelihood of multifactorial genesis of dizziness increases with age, (ii) patients suffered from diseases that could be followed by dizziness (however, patients with non-vestibular migraine were included – two patients suffered from migraine attacks in the past, three patients still had sometimes migraine attacks), (iii) history taking or physical examination revealed an inner ear hearing loss, (iv) dizziness evolved after head trauma, (v) history taking, clinical examination or caloric testing revealed a current or past vestibular disorder of peripheral or central origin, or a cardiovascular dysfunction (e.g., in patients reporting a circumscribed motion illusion a vestibular origin was suspected and the patients were excluded), and (vi) physical examination revealed an objective stance or gait deficit. The detailed reasons for excluding 184 out of 199 patients consecutively examined in our dizziness unit are listed in Figure 2. All patients included underwent a psychiatric assessment (M.F.) and were classified according to ICD-10 and following the classification by Eckhardt-Henn et al. for somatoform vertigo (8).

Twenty-one healthy subjects served as controls for questionnaires and experiments (10 female, 11 male; mean age 35.3 years, range 22–65). Healthy subjects were interviewed on their medical history and complaints of dizziness. If they suffered from dizziness at present or in the past, if they reported a psychiatric disorder at present or if their reports revealed any of the exclusion criteria as described for patients they did not participate in this study.

All patients and healthy subjects gave informed consent in accordance with the Declaration of Helsinki. The study was approved by the local ethics committee of the medical faculty of the University of Tübingen.

### Questionnaires

The severity of dizziness and the psychopathologic burden were assessed by several questionnaires in patients and healthy controls as specified below. This standardized procedure allowed the comparison with previously described characteristics of patients with chronic idiopathic dizziness.

A German translation of the Structured Inventory of Malingered Symptomatology (SIMS) was applied in patients and healthy controls to detect subjects whose answers were suspicious of simulation or aggravation of complaints (16, 17). In subjects with a test value of >16 (one patient) the other questionnaires were not analyzed. A German translation of the Vertigo Symptom Scale (VSS) composed of two subscales was used to assess the extent of symptoms of dysbalance (Vertigo subscale, VER) and accompanying Anxiety and Autonomic symptoms (AA) (18, 19). The Dizziness Handicap Inventory (own German translation) served as a measure for the impact of dizziness on daily life and was therefore only applied in patients but not in healthy controls (20). An own German translation of the Situational Characteristics Questionnaire (SitQ) provided information about the extent of space and motion discomfort (SMD-2) and the discomfort in agoraphobic situations (Ag-1) (4). The Symptom Check List (SCL-90-R) screened for psychopathology (German version, Beltz Test GmbH, Göttingen, Germany). The Beck-Depression-Inventory (BDI-I) was used to assess signs of depression (German version, Hans Huber Verlag, Bern, Switzerland). The State-Trait-Anxiety-Inventory (STAI) tries to contrast state anxiety (scale X1) against trait anxiety (scale X2) (German version, Beltz Test GmbH). The Freiburger Personality Inventory (FPI-R) was applied in order to obtain information about certain personality patterns potentially related to chronic idiopathic dizziness (Hogrefe Verlag, Göttingen, Germany).

### Experiments

Visual acuity was measured with Snellen charts under the same conditions (corrected or uncorrected) as during the experiments. There was no difference between patients and healthy controls (0.63 vs. 0.69, *p* = 0.73).

Psychophysical experiments were carried out in a dark room with the subject sitting 60 cm in front of a screen (120 cm × 160 cm). Stimuli were back-projected onto a transluminant screen. The head was fixed by supporting the forehead with a bar and by a bite bar. The movements of one eye were recorded by means of an infrared camera-based eye tracking system (Chronos, Berlin, Germany) with a sampling rate of 200 Hz. Eye movement calibration was based on asking subjects to sequentially fixate a set of nine target position, representing the corners of an invisible 10° × 30° rectangle, centered on straight ahead as well as its center. For the generation of stimuli, the control of the experiments as well as data acquisition we deployed an open-source software package (NREC, http://nrec.neurologie.uni-tuebingen.de) running under Linux on a standard PC.

**Figure 3:**
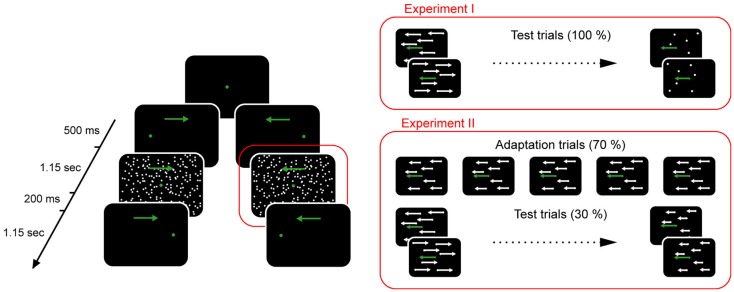
**Psychophysical paradigm deployed to measure the background velocity perceived as being stationary during horizontal smooth pursuit eye movements**. The sequence of events in a given trial is shown on the left side. The green dot represents the target whose slow motion is indicated by a green arrow. A white random dot kinematogram (called background here) was presented around the time the target dot passed the center of the screen. Trials differed with respect to the velocity of the background as illustrated on the right side. Only the two staircase procedures for leftward motion of the target are depicted here. Based on the subjects’ responses the staircase procedures converged on the background velocity perceived as being stationary (point of subjective stationarity). In Experiment II adaptation trials with a constant background velocity in the direction of target motion were added and interleaved with test trials. The addition of adaptation trials leads to a shift of the point of subjective stationarity in the direction of the adaptation trials. See Section “Materials and Methods” for further details.

The psychophysical experiments aimed at assessing the normal predictions of the visual consequences of smooth pursuit eye movements and, in addition, at the adaptation of these predictions by presenting background velocities that deviate from normal in the majority of trials. Smooth pursuit eye movements were elicited by a visual target dot (green, radius = 0.3°) which was initially presented in the middle of the visual field. After a fixation period of 500 ms it jumped pseudorandomly to the left or right by 15° and immediately started to move to the opposite side with a constant velocity of 12°/s spanning a visual angle of 30°(Figure 3). Subjects were instructed to pursue the target dot. During pursuit eye movement, beginning 100 ms before the target dot reached the center of the visual field, a background consisting of 10,000 white dots (radius = 0.2°, 140° horizontal, and 110° vertical extension), plotted in random locations was presented for 200 ms. The white dots moved coherently with constant horizontal angular velocity. Dots disappearing on one side were replotted at the same vertical position on the opposite side. Subjects had to report by button press (left or right) whether they perceived the background moving to the left or right (two-alternative-forced-choice). If the subject fixated the target dot appropriately (the eye had to stay within a fixation window centered on the target during the initial fixation period and during the time of background presentation) and pressed the response button within 1.5 s after background presentation, the trial was considered “valid,” stored for later in depth analysis and rewarded by providing acoustic feedback. In case of inappropriate eye movements the trial was aborted. The additional experimental effort of studying background motion perception during pursuit in alternating directions instead of just one was accepted to rule out a role of possible motion adaptation (“motion aftereffect”).

The background velocity is the key experimental variable that needs to be manipulated in order to assess the prediction of the visual consequences of the smooth pursuit eye movements. The optimal prediction equals the retinal image motion arising from the eye movement made and would therefore result in the perception of a stationary background. A prediction which is too small will lead to the perception of illusory motion of the surrounding in a direction opposite to the direction of the pursuit eye movement [a “normal” Filehne illusion, Ref. (21)]. Conversely, if it is too large visual motion will be perceived in the direction of the pursuit eye movement (“inverted” Filehne illusion). In any case, the background will be perceived as stationary if its physical speed evens out the Filehne illusion. This happens if the background velocity equals the Filehne illusion in size but is opposed to it in direction. We determined this “point of subjective stationarity” (PSS) by varying background velocity according to PEST staircase procedures which converges on the PSS, characterized by equally probable right and left answers (22). A positive PSS indicates that background motion in the direction of pursuit needs to be presented to render the background stationary. This implies that the prediction of the retinal image motion induced by the pursuit eye movement is too small to fully compensate the pursuit-induced Filehne illusion. A negative PSS signifies that the prediction is too large, i.e., it overcompensates pursuit-induced retinal motion, corresponding to an inverted Filehne illusion. Finally, in the case of perfect prediction, the PSS is 0 as no external background motion is needed to perceive the background stationary. The “gain of prediction” (in short “gain”) is 1 in this case, larger than 1 if the pursuit is overcompensated and smaller than 1 if the prediction is too small. It is calculated as follows:

$$\begin{array}{llll} & \text{gain of prediction}=\frac{\text{pursuit velocity}-\text{background velocity at PSS}}{\text{pursuit velocity}}\; & & \; \end{array}$$

**Figure 4:**
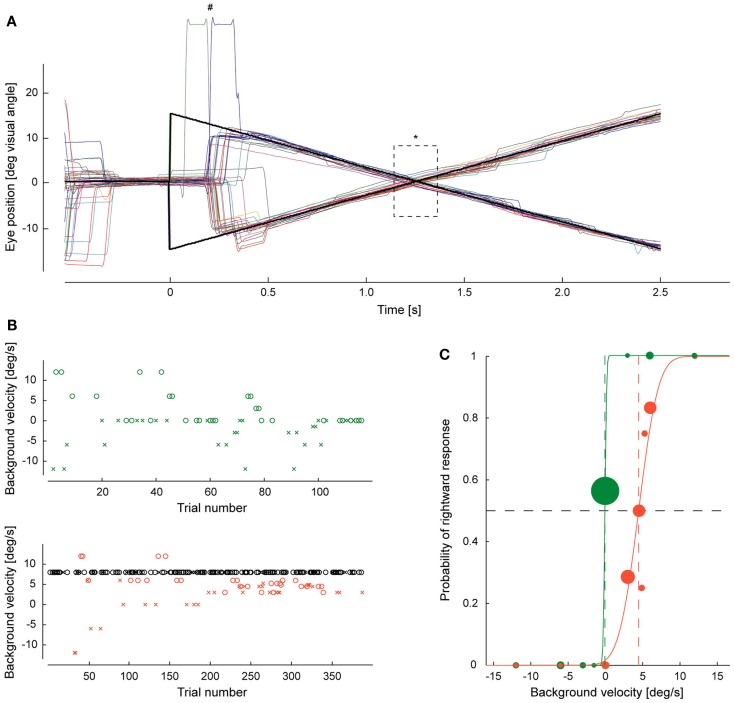
**Example of eye movements and response behavior**. **(A)** Traces of pursuit eye movements (individual trials distinguished by different colors) evoked by step-ramp motion of the target. At time zero the target dot (black) jumps to the right or left followed by a constant movement to the opposite side. ^#^Indicates two eye lid blinking artifacts, *the 200-ms period when the background was presented. **(B)** A subject’s decisions on the direction of background motion, varied according to PEST strategies are plotted over trial number for rightward target motions for Experiment I (upper plot with green symbols) and Experiment II (lower plot with red and black symbols). Red and green symbols signify test trials, black symbols randomly interleaved adaptation trials. “o” marks a perception of background motion to the right, “x” to the left. In Experiment I they converged on a background velocity close to zero. In Experiment II, the convergence zone was shifted toward the velocity of the constant velocity trials. **(C)** The probability of rightward decisions is plotted as function of background velocity for both experiments. The size of the dots corresponds to the number of trials for a given background velocity. The plots are fitted by probit functions whose turning point indicates the point of subjective stationarity, i.e., the background velocity at which subjects vote for right or left at chance level (0.5). Note that the introduction of the constant velocity trials in Experiment II shifted the point of subjective stationarity from about 0°/s to almost 5°/s in this subject.

In Experiment I the gain was determined for both pursuit directions. For each direction, background velocity was varied according to two independent and interleaved PEST strategies, whose starting velocities were of equal size but oppositely directed (12 and −12°/s respectively, Wald constant 1.0). This was necessary in order to avoid subtle influences of the choice of the starting velocity on the PSS (14). The two pursuit directions and the associated 2 × 2 PEST strategies controlling background velocity were presented pseudorandomly until 30 valid trials for each of the four conditions had been collected (Figure 4).

Experiment II and control Experiment III were conducted in order to assess the ability to adapt the gain predicting the visual consequences of pursuit eye movements. The motivation to test for gain adaptation in patients is based on the observation that healthy subjects exhibit decreased gains if being exposed to long series of pursuit trials in which the background is moving consistently in the direction of the eye movement. This configuration is initially associated with the perception of background motion in the direction of eye movement. As the visual system obviously prefers the ecologically plausible interpretation that consistent motion of the visual world, simulated by the background motion, must be a consequence of judging with wrong, here too large predictions, the gain needs to be changed, which means downregulated here. As a consequence the initially perceived background motion in the direction of the eye movement is reduced and a newly, illusory perception opposite to the direction of the eye movement occurs if the visual system is all of a sudden challenged by pursuit across a stationary background. This gain adaptation was studied here by modifying the paradigm used in Experiment I as follows: in addition to trials whose background velocities were controlled by the aforementioned four PEST strategies, needed to gage the gain, we added a large fraction (70%) of trials in which the same, constant background velocity (8°/s), always in direction of the pursuit, was presented. Independent of pursuit direction a background motion in the direction of the pursuit is initially perceived as if the gain was too high. As a consequence, we expected a downregulation of the gain over the course of the experiment.

In Experiment III, the trials with constant background velocity in pursuit direction were replaced by trials in which the background was actually stationary in the world (i.e., 0°/s), hence not causing unpredicted retinal image motion. The purpose of this experimental manipulation was to control for the difference in duration of Experiments II and I and potential unspecific strategies prompted by the frequent presentation of trials with the same, constant appearance of the background, unrelated to its velocity. The first 20 trials in Experiment II were always trials with 8°/s background velocity to allow some adaptation to start. Correspondingly, in control Experiment III, the first 20 trials were stationary background trials. After this initiation phase, PEST trials and trials with the constant background stimuli were presented pseudorandomly interleaved until a total of 120 PEST trials and 280 constant trials had been collected (Figure 4).

### Data analysis and statistics

Eye movement and response data stored in hdf5 format were analyzed using MATLAB R2009b. The median velocity of horizontal pursuit eye movement per trial was determined for the 200-ms of background presentation, excluding sample points in which the eye velocity record was determined by catch up saccades or influenced by blinks or obvious artifacts. Thereafter, the median pursuit velocities across trials per experiment and direction were calculated for each subject.

Separately for each experiment and pursuit direction, the responses to background velocities controlled by the two independent PEST strategies were pooled. If the responses could be significantly (5% level) approximated by a probit function, the PSS and the just noticeable difference (JND) were extracted (23). The JND corresponds to the width of the probit function and is defined as the difference between the background velocities prompting 25 and 75% decisions in favor of perceived background motion in the direction of pursuit. The JND indicates how precisely a subject differentiates background velocities during smooth pursuit eye movements and it is influenced by the subject’s motivation and cooperation. We used the JND as an inclusion criterion for further analysis: the JND had to be below or equal to 12°/s implying that the subject was able to detect the retinal image motion induced by the pursuit eye movement at all. For each subject we collected the PSS, the JND, and the median pursuit velocity per direction and experiment. In order to justify pooling of data across directions we compared these measures between the pursuit directions separately for each experiment and group by the non-parametric Wilcoxon signed rank test. As we did not find any significant difference between directions at the corrected 5% level, we pooled the data across directions for the main comparisons of PSS, JND, and pursuit velocities between patients and healthy controls.

For statistical comparisons of experimental data and questionnaires between patients and healthy subjects the non-parametric Wilcoxon rank-sum test was used. For comparisons between experiments within both groups the non-parametric Wilcoxon signed rank test was applied (significance level 5%). Bonferroni-correction for multiple comparisons was performed as specified. Correlation coefficients were tested for significance with *t*-statistics. Group values are given as mean ± SEM. In order to weight how good certain scales of questionnaires discriminate between patients and healthy controls we conducted a receiver operating characteristics analysis (ROC). The area under the curve of the ROC was used as a measure for the discrimination strength between patients and healthy controls. All statistics were calculated in MATLAB R2009b.

## Results

Chronic idiopathic dizziness is a concept that is able to embrace different previously suggested clinical entities that do not always emphasize the same symptoms and diagnostic criteria. To allow the comparison of our patient group with these different entities we describe disease symptoms, triggers, and time courses of our patients in detail and quantify them by questionnaires as far as possible.

### Clinical characterization of patients

Out of the five supplied terms describing the character of their dizziness most patients (8 out of 15) affirmed four of them, 4 patients all five and 3 patients only three. Actually, no patient had to be excluded for not affirming at least three of the terms (Table 1). As depicted in Table 2, all except one patient had dizziness of fluctuating intensity reaching the level of attacks in four patients. Ten patients did not experience any symptom-free interval and two patients had no symptom-free moment for days. Illusory body perturbations described as lasting fractions of seconds were perceived by seven patients. On average disease duration was 2–3 years (mean: 31.6 months, range: 4–84 months, one outlier of 47 years). Dizziness was triggered or augmented by visual motion stimuli, specific environments, or social situations in four, three, and three patients, respectively. An avoidance behavior was developed by six patients. An improvement of dizziness by alcohol intake, sports, or distraction was reported by 2, 5, and 10 patients, respectively. Only one patient, who did not suffer from attacks, denied any avoidance behavior, and any influence on dizziness by these factors. Anxiety accompanied by dizziness was experienced by 10 patients, the anxiety to fall by 7 patients. As a consequence of the exclusion criteria none of the patients had an onset of dizziness that was suspicious for a vestibular origin. The onset of dizziness was associated with a circumscribed life event in three patients, a prior non-vestibular illness in two patients, and augmented private or occupational stress in four patients. No initial trigger could be gleaned in seven patients. Altogether, 13 of 15 patients could be diagnosed as suffering from PPV according to the diagnostic criteria of Huppert et al. (24).

**Table 1:** **Character of dizziness as indicated by the patient’s answers to five expressions related to the experience of dizziness**.

No	Feeling of swaying	Light-headedness	Unsteadiness standing/walking	Wrong in head	Disturbed balance
1	Yes	Yes	Yes	Yes	Yes
2	Yes	Yes	No	Yes	No
3	No	Yes	Yes	Yes	No
4	Yes	Yes	Yes	Yes	No
5	No	Yes	Yes	Yes	Yes
6	Yes	Yes	Yes	Yes	No
7	Yes	Yes	Yes	Yes	Yes
8	Yes	Yes	Yes	No	Yes
9	Yes	Yes	Yes	No	Yes
10	No	No	Yes	Yes	Yes
11	Yes	No	Yes	Yes	Yes
12	Yes	Yes	Yes	Yes	Yes
13	No	Yes	Yes	Yes	Yes
14	No	Yes	Yes	Yes	Yes
15	Yes	Yes	Yes	Yes	Yes

**Table 2:** **Clinical characteristics of patients suffering from chronic idiopathic dizziness**.

No	Gender	Age in years	Duration in months	Frequency in days per month	Sustained or episodic	Fluctuating or attacks	First trigger	Psychiatric diagnosis
1	Male	45	15	8–13	Episodic	Attacks	No	Dissociative disorder
2	Male	28	48	Daily	Sustained	Attacks	Stress, illness	Panic disorder
3	Female	40	56	Daily	Sustained	Attacks	Childbirth	Panic disorder
4	Female	20	4	Daily	Sustained	Attacks	No	Recurrent depressive disorder
5	Male	69	564	Daily	Sustained	Fluctuating	No	Somatization disorder
6	Female	42	5	Daily	Sustained	Attacks	Death father	Severe depressive episode
7	Male	48	36	Daily	Sustained	Attacks	stress	Somatization disorder
8	Female	30	66	Daily	Sustained	Fluctuating	Driving license	Somatization disorder
9	Male	31	54	Daily	Episodic	Fluctuating	Stress	Somatization disorder
10	Female	27	13	8	Sustained	Fluctuating	Stress	Somatization disorder
11	Female	51	6	Daily	Sustained	Attacks	No	Generalized anxiety disorder
12	Female	37	9	10–15	Sustained	Fluctuating	No	Moderate depressive episode
13	Male	51	60	8–10	Episodic	Fluctuating	No	Panic disorder
14	Female	22	26	Daily	Sustained	Fluctuating	Illness	Panic disorder with agoraphobia
15	Male	30	21	Daily	Sustained	Attacks	No	Dissociative disorder

The frequency of dizziness is the number of days patients suffered from dizziness. Whether or not dizziness was with or without symptom-free intervals on these days is indicated by “Episodic” or “Sustained.” Attack denotes the sudden emergence of dizziness or its sudden increase experienced by patients at least occasionally. The psychiatric diagnosis given is considered the clinically most likely one related to dizziness following the concept of Eckhardt-Henn et al. (8).

All patients were interviewed by a Psychiatrist (M. F.) and assessed according to ICD-10 and Eckhardt-Henn et al. (8). Most patients revealed psychopathologic conditions which could, following the concept of primary somatoform dizziness, be responsible for dizziness. Out of those, three patients had a panic disorder, one patient agoraphobia with panic disorder, one patient a generalized anxiety disorder, two patients a dissociative disorder, two patients a depressive episode, and one patient a recurrent depressive disorder. In one third of the patients an additional psychopathologic condition putatively responsible for the complaints of dizziness could not be found. In these cases a somatization disorder was diagnosed (Table 2).

### Questionnaires confirm that patients primarily suffer from situation-dependent dizziness accompanied by anxiety

**Figure 5:**
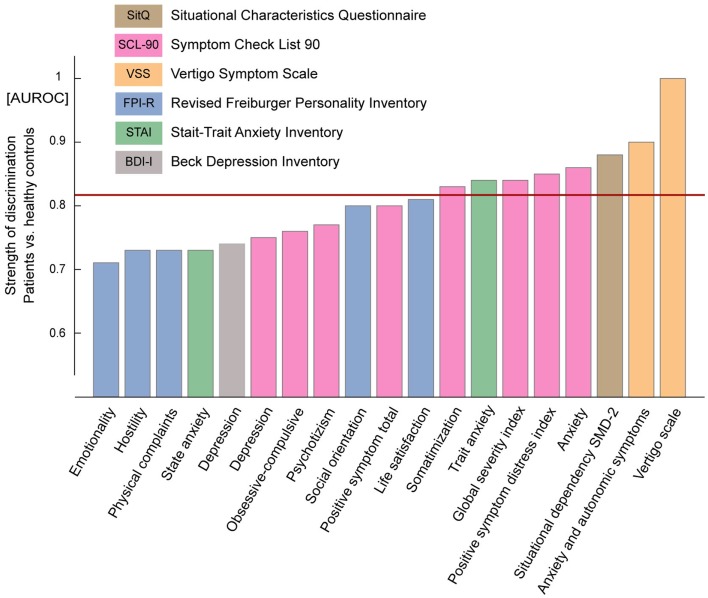
**Summary of the results of the questionnaires**. Questionnaires are distinguished by colors. Subscale names are given below the columns. Only those subscales that showed a difference above the 5% significance level between patients and healthy controls are depicted here. The values plotted are AUROC-values which quantify the strength of discrimination between patients and healthy controls. Note the ideal discrimination of 1.0 for the VER subscale of the VSS measuring dizziness. Particular strong discriminations were observed for most anxiety subscales and the situational dependency of complaints. A value of 0.5 is the lowest possible value indicating no discrimination between groups. The horizontal red line indicates the threshold for significant differences after Bonferroni-correction for 31 comparisons.

The psychopathologic symptom pattern common to several concepts related to chronic idiopathic dizziness was reconfirmed by comparing the results of questionnaires between the patient group and healthy subjects as depicted in Figure 5. The values plotted reflect the area under the curve describing the Receiver Operating Characteristics which represent the strength of discrimination for each subscale. This illustration summarizes which features distinguish the patient group from healthy subjects and it weights these features with respect to each other assuming that the questionnaires are equally valid. An optimal discrimination of 1.0 was achieved by the VER subscale of the VSS, which is an expected result indicating that patients suffer from dizziness but healthy subjects do not. This result confirms the validity of the VER for chronic idiopathic dizziness. The most prominent characteristics of the patients that remained significant after Bonferroni-correction for 31 comparisons were anxiety, in particular the anxiety trait, and the situational dependency of dizziness. Two global measures of psychopathologic burden and the somatization subscale of the SCL-90 showed a similar extent of discrimination. The test values of each subscale and their statistical comparisons between patients and healthy subjects are provided in Table 3.

**Table 3:** **Mean and standard error of the mean (SEM) of test values of all subscales (for SCL-90 *t*-values are used) are listed for patients and healthy controls**.

Questionnaire with subscale		Healthy subjects		Patients		Difference
		Mean	SEM	Mean	SEM	*p*
VSS	VER	0.07	0.01	0.65	0.06	0.0000009
	AA	0.56	0.11	1.68	0.18	0.0001
DHI	DHI	NaN	NaN	32.30	3.20	NaN
SitQ	SMD-2	2.41	0.35	6.70	0.98	0.0001
	Ag-1	3.94	0.62	4.37	1.12	0.94
BDI-I	BDI-I	3.62	0.81	8.00	1.51	0.02
STAI	X1	32.00	1.20	37.00	1.80	0.02
	X2	32.50	1.60	44.10	2.60	0.001
SCL-90	Somatization	45.38	2.10	58.93	2.12	0.001
	Obsessive-compulsive	49.43	1.74	57.71	2.33	0.01
	Interpersonal sensitivity	48.05	1.57	54.50	2.76	0.06
	Depression	49.48	1.56	57.93	2.88	0.02
	Anxiety	47.48	1.55	57.71	1.84	0.0004
	Hostility	47.14	1.95	54.71	2.73	0.03
	Phobic anxiety	49.71	1.31	56.07	2.83	0.09
	Paranoid ideation	48.38	1.79	49.86	3.14	0.95
	Psychoticism	47.95	1.52	56.14	2.45	0.01
	GSI	48.14	1.54	57.93	2.24	0.001
	PST	48.67	1.54	56.86	1.94	0.003
	PSDI	48.57	0.88	56.86	2.14	0.001
FPI-R	Life satisfaction	6.10	0.43	3.71	0.47	0.002
	Social orientation	6.71	0.32	4.86	0.39	0.002
	Achievement orientation	4.81	0.38	4.79	0.57	0.97
	Restrainingness	4.33	0.35	5.50	0.44	0.05
	Exciting harness	4.05	0.37	4.64	0.52	0.43
	Aggressiveness	3.67	0.47	4.64	0.51	0.07
	Demand	4.19	0.46	5.29	0.59	0.17
	Physical complaints	4.10	0.33	5.57	0.47	0.02
	Health concerns	4.62	0.34	4.57	0.39	0.96
	Openness	5.38	0.54	5.07	0.54	0.65
	Extraversion	4.90	0.38	4.64	0.43	0.64
	Emotionality	3.95	0.46	5.57	0.52	0.04

The *p*-value of the non-parametric Wilcoxon rank-sum test is depicted in the last column. Subscales given in red revealed a significant difference between patients and healthy subjects at the uncorrected level of 5%. VSS, vertigo symptom scale; VER, vertigo subscale; AA, anxiety and autonomic symptoms subscale; DHI, dizziness handicap inventory; SitQ, situational characteristics questionnaire; SMD-2, space and motion discomfort; Ag-1, discomfort in agoraphobic situations; BDI-I, beck-depression-inventory, first version; STAI, state-trait-anxiety-inventory; X1, state subscale; X2, trait subscale; SCL-90, symptom check list; GSI, global severity index; PST, positive symptom total; PSDI, positive symptom distress index; FPI-R, Freiburger personality inventory, revised version.

### Patients are not impaired in the prediction of the visual consequences of smooth pursuit eye movements

**Figure 6:**
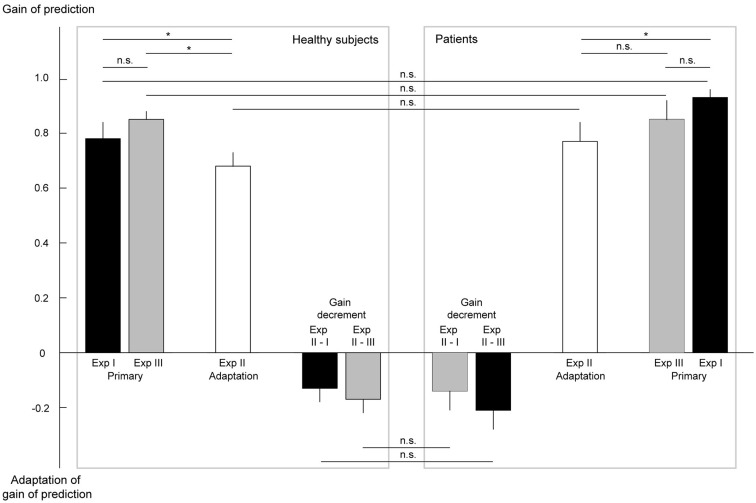
**Results of the psychophysical experiments addressing the prediction of pursuit-induced retinal image motion and its adaptation**. The gains of prediction of eye movement-induced background motion are plotted. A gain below 1.0 reflects an underestimation of self-induced retinal image motion corresponding to a Filehne illusion. A gain of larger than 1.0 (not observed) would indicate overestimation or an inverted Filehne illusion. In the experiments in which the native, non-adapted sensory prediction was determined (Experiments I and III), the gain was not different between patients and healthy subjects and, moreover, clearly smaller than one. Independent of whether or not trials with stationary background were presented (Experiments III vs. I) the gains were the same, in patients as well as in healthy subjects. In Experiment II, both groups showed a significant adaptation of their gain with respect to the gain in Experiment I. The amount of adaptation (gain decrement) did not differ between patients and healthy controls. Error bars indicate the standard error of the mean. A non-significant difference is indicated by n.s., a significant one by *.

The first experiment was deployed to assess whether patients have difficulties in predicting the visual consequences of smooth pursuit eye movements. If there was a lack of prediction one would expect a shift of the gain of prediction toward lower values. However, we measured no statistical difference between patients (mean 0.93 ± 0.03 SEM) and healthy subjects (0.78 ± 0.06; *p* = 0.13, *n* = 12, *n* = 20; see Figure 6). This result was confirmed by the third experiment which contained 70% zero velocity trials (patients: 0.85 ± 0.07, *n* = 12 and healthy subjects: 0.85 ± 0.03, *n* = 19, *p* = 0.53). In both experiments, patients as well as healthy subjects exhibited gains below 1.0, reflecting a small underestimation of the pursuit-induced retinal image motion, leading to a Filehne illusion. The statistical comparison of the gains for the first and the third experiment revealed no difference, neither for patients nor for healthy controls (*p* = 0.37, *p* = 0.45). This lack of a difference demonstrates that the additional presentation of several hundred constant trials by itself does not confound the gain of prediction.

### Patients adapt their prediction of the visual consequences of smooth pursuit eye movements adequately

In view of the normal ability to predict the visual consequences of pursuit eye movements in patients one could argue that disturbances in prediction become only unmasked if the system is challenged by the need to adjust the prediction to the needs of the situation. Actually, this may also be suggested by frequent reports of dizziness being triggered by complex visual scenes. To reveal potentially inadequate adaptation to new demands, we conducted the second experiment, in which trials with constant background motion of 8°/s in the direction of pursuit were added. As explained earlier, in healthy subjects these stimuli should induce a decrease of the gain. Indeed, this was what we found in healthy subjects (0.68 ± 0.05, *n* = 19, *p* = 0.020). However, contrary to the hypothesis of impaired gain adaptation in patients we obtained a similar decrease in the patient group (0.77 ± 0.07, *n* = 14, *p* = 0.021). Not only the absolute gain values were the same in both groups but also the individual changes of gain values when comparing the adapted gain in Experiment II with the primary gains in Experiments I or III (Figure 6).

As already mentioned, the experience of illusory body perturbations might be seen as a particular clear sign of a disturbed reafference mechanism. About half of our patients reported such an illusion. When comparing this subgroup to the one without illusory body perturbations, we likewise could not find any significant deviation, neither of their absolute gain in Experiment I, nor in the gain change they exhibited with respect to Experiment I (first experiment, 0.93 ± 0.05 vs. 0.94 ± 0.05, second experiment – first experiment adaptation −0.22 ± 0.11 vs. −0.20 ± 0.10).

Normal measures of predictive behavior in patients go hand in hand with completely normal performance with respect to any other aspect of the task we could conceive of. First of all, the pursuit velocities did not differ significantly between groups (first experiment: 11.7 ± 0.1 vs. 10.9 ± 0.4°/s, second experiment: 11.1 ± 0.2 vs. 10.7 ± 0.4°/s, and third experiment: 11.2 ± 0.3 vs. 10.5 ± 0.4°/s). Secondly, subjects’ cooperation as captured by the JND (see Materials and Methods) did not differ between patients and healthy subjects (first experiment: 5.8 ± 0.7 vs. 5.6 ± 0.4°/s, second experiment: 5.8 ± 1.0 vs. 5.8 ± 0.4°/s, and third experiment: 4.9 ± 0.9 vs. 5.1 ± 0.6°/s).

### The prediction of eye movement-induced retinal motion is not related to the severity of dizziness or psychopathological burden

Finally, we addressed the question if the prediction of the visual consequences and its adaptation correlated with the severity of dizziness or certain psychopathological findings measured by questionnaires. In fact, none of the correlation coefficients calculated exhibited significance after correction for multiple comparisons (Table 4).

**Table 4:** **Correlation coefficients and *p*-values for correlations between all subscales of all questionnaires and the primary gain of prediction and the amount of adaptation, respectively (negative correlation for adaptation indicates stronger adaptation, i.e., larger gain decrement, with increasing values on the subscale) are shown separately for healthy subjects, patients, and both groups pooled**.

Questionnaire with subscale		Healthy subjects				Patients				Pooled			
		Primary		Adaptation		Primary		Adaptation		Primary		Adaptation	
		*r*	*p*	*r*	*p*	*r*	*p*	*r*	*p*	*r*	*p*	*r*	*p*
VSS	VER	0.07	0.76	−0.09	0.72	0.21	0.55	0.15	0.66	0.32	0.07	−0.25	0.18
	AA	0.29	0.21	−0.30	0.22	−0.31	0.36	0.06	0.86	0.32	0.08	−0.31	0.09
DHI	DHI	NaN	NaN	NaN	NaN	−0.22	0.52	−0.60	0.052	NaN	NaN	NaN	NaN
SitQ	SMD-2	0.11	0.63	−0.05	0.84	0.15	0.67	−0.54	0.09	0.28	0.13	−0.41	0.02
	Ag-1	−0.08	0.73	0.15	0.54	0.06	0.85	−0.21	0.53	0.00	0.99	−0.04	0.83
BDI-I	BDI-I	0.55	0.012	−0.39	0.10	−0.25	0.46	−0.40	0.22	0.40	0.03	−0.46	0.010
STAI	X1	0.04	0.88	0.45	0.053	−0.16	0.65	−0.27	0.42	0.17	0.36	0.01	0.95
	X2	0.12	0.62	−0.14	0.57	−0.11	0.74	−0.31	0.36	0.26	0.15	−0.34	0.06
SCL-90	Somatization	0.25	0.29	0.05	0.85	−0.05	0.89	−0.51	0.11	0.35	0.05	−0.28	0.14
	Obsessive-compulsive	0.02	0.94	−0.28	0.24	−0.32	0.34	−0.59	0.055	0.12	0.53	−0.48	0.0079
	Interpersonal sensitivity	−0.09	0.70	−0.06	0.82	−0.24	0.47	−0.17	0.61	0.02	0.92	−0.21	0.27
	Depression	0.15	0.54	0.11	0.65	−0.43	0.18	−0.52	0.10	0.13	0.50	−0.29	0.12
	Anxiety	0.14	0.54	−0.17	0.50	−0.27	0.43	−0.15	0.66	0.24	0.18	−0.30	0.10
	Hostility	0.02	0.92	−0.11	0.64	−0.42	0.19	−0.44	0.18	0.07	0.69	−0.33	0.07
	Phobic anxiety	−0.16	0.50	0.08	0.76	−0.03	0.93	−0.22	0.52	0.05	0.80	−0.19	0.32
	Paranoid ideation	−0.28	0.24	−0.18	0.46	−0.48	0.14	−0.14	0.69	−0.22	0.23	−0.20	0.29
	Psychoticism	0.15	0.52	−0.21	0.39	−0.23	0.50	−0.50	0.12	0.21	0.26	−0.42	0.02
	GSI	0.08	0.72	−0.09	0.70	−0.42	0.20	−0.46	0.15	0.16	0.38	−0.36	0.051
	PST	0.02	0.93	−0.11	0.65	−0.38	0.25	−0.36	0.28	0.12	0.51	−0.32	0.08
	PSDI	0.39	0.09	−0.10	0.69	−0.23	0.49	−0.50	0.12	0.31	0.09	−0.41	0.03
FPI-R	Life satisfaction	−0.36	0.11	0.28	0.25	0.76	0.007	−0.06	0.86	−0.32	0.08	0.30	0.11
	Social orientation	0.20	0.39	−0.13	0.60	0.22	0.51	−0.13	0.71	0.03	0.89	0.02	0.91
	Achievement orientation	−0.14	0.56	0.09	0.71	0.35	0.30	0.24	0.49	0.00	0.99	0.10	0.58
	Restrainingness	−0.19	0.42	−0.28	0.24	−0.45	0.16	0.10	0.77	−0.07	0.72	−0.25	0.19
	Exciting harness	0.07	0.78	0.00	1.00	−0.54	0.09	0.43	0.18	0.03	0.88	0.09	0.63
	Aggressiveness	−0.14	0.55	−0.24	0.33	−0.51	0.11	0.11	0.75	−0.12	0.53	−0.17	0.36
	Demand	0.03	0.89	−0.13	0.58	−0.42	0.20	−0.62	0.04	0.06	0.75	−0.39	0.03
	Physical complaints	0.28	0.22	0.11	0.66	−0.53	0.10	0.38	0.24	0.26	0.15	0.02	0.90
	Health concerns	−0.32	0.18	0.11	0.66	0.36	0.28	0.13	0.71	−0.18	0.34	0.08	0.68
	Openness	−0.07	0.77	0.01	0.97	−0.32	0.33	0.28	0.41	−0.11	0.54	0.11	0.58
	Extraversion	−0.02	0.94	0.10	0.69	−0.11	0.76	−0.19	0.57	−0.03	0.86	0.00	0.99
	Emotionality	−0.07	0.75	0.02	0.94	−0.56	0.08	−0.30	0.37	0.00	0.98	−0.19	0.32

Red subscales are the ones that gave different scores for patients and healthy controls at the uncorrected level of 5% (taken from the results depicted in Table 3). Numbers given in red are correlations significant at the uncorrected level of 5%.

## Discussion

The aim of this study was to critically test the hypothesis that the sensory predictions of self-motion are malfunctioning in patients suffering from chronic idiopathic dizziness. An inappropriate prediction of the sensory consequences of one’s own movements would lead to an instable perception of the stationary world, subjectively experienced as dizziness when moving. We tested this hypothesis resorting to the study of the perception of visual motion during smooth pursuit eye movements as a model of a more general principle. Our results do not support the assumption of an altered sensory prediction. The patients predicted retinal image motion induced by smooth pursuit eye movements in the same way as healthy subjects. Moreover, they exhibited the same adaptation of their predictions according to the needs of the visual context. Finally, the individual deviations from ideal predictions did not correlate with any of the measures used to assess the severity of dizziness or psychopathological burden.

In an attempt to gage predictions of the sensory consequences of body movements, we studied horizontal smooth pursuit eye movements as a model of motor behavior. The choice of this particular model was suggested by the rich experience gained in similar experiments on healthy subjects as well as patients with varying problems in the past (14, 15, 25–27). The present study confirms our previous results demonstrating an underestimation of the prediction of pursuit eye movement-induced retinal image motion under laboratory conditions as first described by Filehne (21). Moreover, the amount of adaptability of this prediction found in this and previous studies is very similar. Could it be that the absence of a significant difference between the two groups is simply a consequence of insufficient sensitivity of the psychophysical measurements? We think that this is unlikely, given the fact that two previous studies from our laboratory aiming at different types of patients yielded clear deviations from healthy subjects (15, 26). This demonstrates that the method used is, at least in principle, capable to detect pathologic alterations of pursuit related sensory predictions.

However, it remains open if pursuit related sensory stimulation is indeed a valid generic model of movement related sensory predictions. One might argue that alterations of sensory predictions relevant for dizziness might occur with respect to one type of motor behavior – and not necessarily smooth pursuit – but not affect others. In other words, when studying smooth pursuit eye movements we may have simply missed the relevant motor behavior. Actually, patients with chronic idiopathic dizziness often relate their dizziness to head movements and, moreover, they report an increase in dizziness when making head movements during walking compared to standing still (6). Although this may at first glance suggest a specific role of head movement and walking related sensory predictions, one should keep in mind that any head movement is accompanied by stabilizing smooth eye movements, which may well be the aspect of the movement repertoire, relevant for the occurrence of dizziness.

In the present study we focused on patients with chronic idiopathic dizziness which we defined pragmatically by the non-vertiginous character of their dizziness, a time criterion, and negative criteria minimizing the likelihood of a classic organic disease. This approach was motivated by the inconsistent state of the literature on this issue. Patients with chronic idiopathic dizziness have been reported under the headings of space and motion phobia, PPV, somatoform dizziness, visual vertigo, and chronic subjective dizziness, just to mention the more important ones (5–7, 9, 28). Each concept emphasizes particular symptoms and favors a certain underlying mechanism. However, these various clinical concepts largely overlap, which is why in our view attempts to distinguish clear-cut categories remain artificial. This is why we found it pertinent to describe the patients’ symptoms in detail and to refrain from diagnostic categorizations reflecting one of those particular concepts. Nevertheless, in view of the congruency between the diagnostic criteria of PPV and the symptom pattern of our patients it appears justified to apply our findings to PPV (24, 29). In particular, an additional analysis restricted to the subgroup of patients reporting illusory body perturbations, a symptom which had originally motivated the hypothesis, could also not find deviating predictions of visual consequences. It remains, however, the possibility that certain patients with chronic idiopathic dizziness who specifically report an augmentation of dizziness or oscillopsia during eye movements might suffer from eye movement related disturbances of sensory predictions of self-motion as shown for one patient with bilateral parietal lesions (15).

As an alternative, one might take our failure to reveal impaired sensory predictions in chronic idiopathic dizziness as argument to reconsider disturbances in more peripheral, sensory organ-related information processing. Actually, such a perspective might seem promising in view of the increasing number of dizziness patients shown to suffer from subtle vestibular deficits, hitherto ignored (30–32). A case in point is the description of the superior canal dehiscence syndrome by Minor and colleagues a few years ago (33). Probably, these patients would have been classified as “idiopathic” or “psychosomatic” two decades ago. The observation that many patients with chronic, to date, idiopathic dizziness suffer primarily during walking may point toward disturbances evoked by head movements. Movements of the head require not only vestibulo-ocular reflex dependent, compensatory eye movements but also the suppression of them when the subject overtly shifts attention, i.e., saccades from one object to the other. Hence, it seems a reasonable possibility that such more complex interactions between vestibular input and eye movement control are deficient. Such disturbances may be unraveled if subjects are examined in experimental conditions that require locomotion, the controlled shift of attention but also the measurement of eye, head, and body movements, and optimally, the monitoring of motion perception.

In conclusion, our study falsifies the hypothesis that impaired predictions of the sensory consequences of horizontal smooth pursuit underlie chronic idiopathic dizziness or PPV. However, this negative result does not exclude altered predictions of the sensory consequences of other types of motor behavior such as locomotion, a possibility that needs to be addressed in future experiments.

## Conflict of Interest Statement

The authors declare that the research was conducted in the absence of any commercial or financial relationships that could be construed as a potential conflict of interest.
